# Multivariate Analysis of Fruit Antioxidant Activities of Blackberry Treated with 1-Methylcyclopropene or Vacuum Precooling

**DOI:** 10.1155/2018/2416461

**Published:** 2018-02-01

**Authors:** Jian Li, Guowei Ma, Lin Ma, Xiaolin Bao, Liping Li, Qian Zhao, Yousheng Wang

**Affiliations:** Beijing Advanced Innovation Center for Food Nutrition and Human Health, Beijing Engineering and Technology Research Center of Food Additives, Beijing Technology and Business University, Beijing 100048, China

## Abstract

Effects of 1-methylcyclopropene (1-MCP) and vacuum precooling on quality and antioxidant properties of blackberries (*Rubus* spp.) were evaluated using one-way analysis of variance, principal component analysis (PCA), partial least squares (PLS), and path analysis. Results showed that the activities of antioxidant enzymes were enhanced by both 1-MCP treatment and vacuum precooling. PCA could discriminate 1-MCP treated fruit and the vacuum precooled fruit and showed that the radical-scavenging activities in vacuum precooled fruit were higher than those in 1-MCP treated fruit. The scores of PCA showed that H_2_O_2_ content was the most important variables of blackberry fruit. PLSR results showed that peroxidase (POD) activity negatively correlated with H_2_O_2_ content. The results of path coefficient analysis indicated that glutathione (GSH) also had an indirect effect on H_2_O_2_ content.

## 1. Introduction

Blackberries (*Rubus* spp.) are notable for their antioxidant activities, particularly due to their high contents of polyphenolic compounds, such as ellagic acid, quercetin, gallic acid, anthocyanins, and cyanidins, plus excellent contents of the antioxidant vitamins A and C [1–3]. However, blackberries have a short market life and the fruit quality is rapidly reduced after harvesting [4, 5].

Reactive oxygen species (ROS), like hydrogen peroxide (H_2_O_2_) and superoxide O^*∙*−^, could promote the oxidation of proteins and lipids and thus lead to fruit senescence and a decrease of fruit quality [6, 7]. The antioxidant enzymes, including catalase (CAT), ascorbate peroxidase (APX), peroxidase (POD), and polyphenol oxidase (PPO), can prevent the accumulation of ROS and repair oxidative damage [8]. The antioxidants, such as polyphenolic compounds, also play an important role in scavenging excess ROS [9]. There are many correlated factors affecting the fruit quality. Hence, application of a multivariate technique to characterize the relationship among the antioxidant enzymes' activities, ROS levels, and antioxidant activities seems to be appropriate.

Principal component analysis (PCA) is a multivariate technique used to extract the important information from multivariate data [10]. Partial least squares (PLS) and path analysis can be especially useful to find the correlations between intercorrelated quantitative dependent variables. PLS has been used to predict the changes of quality of pasteurized pineapple juice during storage [11].

In this study, the quality and antioxidant parameters were collected from 1-MCP treated, vacuum precooled, and control fruit. Principal component analysis (PCA) was applied to evaluate the effect of 1-MCP and vacuum precooling on physiological properties of fruit. Correlations between quality and antioxidant parameters were studied through partial least squares (PLS) and path analysis.

## 2. Materials and Methods

### 2.1. Plant Material and Treatment

Blackberry fruit (*Rubus* spp. cv. Triple Crown) at the commercially mature stage was harvested from an orchard near Beijing. Fruit was sorted to eliminate damaged and diseased fruit and selected for uniformity in size and color. Fruit was randomized and divided into 3 lots for the following treatments: (1) untreated and referred to as the control and (2) treated with 5 *μ*g/L 1-methylcyclopropene (1-MCP). Fruits were placed in sealed 250 L plastic chambers with 1-MCP powdered formulation (1250 *μ*g 1-MCP release) at room temperature for 24 h and (3) precooled in vacuum cooler at 10°C for 1 h.

After the treatments, all the fruits were placed in 60 cm × 37 cm × 20 cm plastic containers and then stored at 0°C with 85–95% RH.

### 2.2. Fruit Quality Measurement

Flesh firmness of the fruit was measured using a texture analyzer (LFRA, Brookfield Ltd., USA). Fifteen fruits were measured at each sampling time.

Fruit of each treatment was distributed into three groups (30 fruit per group), and each group represented one replicate. The number of decayed fruits in each plastic container was counted and decay rate was calculated as percentage of decayed fruit versus total fruit.

### 2.3. Enzymatic Activity Analysis

For the ascorbate peroxidase (APX), peroxidase (POD), and polyphenol oxidase (PPO) activities assay, 10.0 g samples were thoroughly homogenized with 20 mL extracting buffer (pH 7.8 100 mM phosphate buffer containing 0.2 g polyvinylpolypyrrolidone) and centrifuged at 10,000 ×g at 4°C for 20 min, and the supernatant was collected and stored at −80°C for further analysis.

APX activity was determined spectrophotometrically by monitoring the decline in absorbance at 290 nm as ascorbate was oxidized [12]. APX activity was expressed as U·g^−1^ FW.

POD activity was measured as the oxidation of guaiacol in the presence of H_2_O_2_ by measuring the absorbance at 460 nm [13]. The POD activity was expressed as U·g^−1^ FW.

PPO activity was measured according to the method of Jiang et al. and expressed as U·g^−1^ FW [13].

### 2.4. GSH Assays

Glutathione (GSH) was extracted from 10.0 g of the flesh tissue with 20 mL of ice-cold 5% trichloroacetic acid containing 5 mM Ethylenediaminetetraacetic Acid (EDTA) and then centrifuged at 4°C for 10 min at 10,000 ×g. The supernatant was assayed for GSH according to the method of Guri, and the GSH content was expressed as mg·100 g^−1^ FW [14].

### 2.5. H_2_O_2_ Assays

H_2_O_2_ was extracted by homogenizing 10.0 g of fruit tissue in 20 ml of cold acetone and was measured according to the method of Brennan and Frenkel [15]. The H_2_O_2_ content was expressed as mg·100 g^−1^ FW.

### 2.6. Antioxidant Activities Assay

For antioxidant activities assay, 10.0 g samples were thoroughly homogenized with 20 mL methyl alcohol and centrifuged at 10,000 ×g at 4°C for 20 min. The supernatant was used for antioxidant activities, total phenol, and total flavonoid concentration assays.

Total antioxidant activity was measured using ferric reducing antioxidant potential assay (FARP) [16]. The values were expressed as the concentration of antioxidants having a ferric reducing ability equivalent to that of 1 mmol/L FeSO_4_.

Trolox equivalent antioxidant capacity (TEAC) was determined according to the method of Arts et al. [17]. 50% of the 2,2′-Azinobis-(3-ethylbenzothiazoline-6-sulphonate) (ABTS) radical-scavenging activity is defined as one activity unit. TEAC activity was expressed as U·g^−1^ FW.

The 2,2-diphenylpicrylhydrazyl (DPPH) radical-scavenging activity was assayed by the method of Shon et al. [18]. 50% of the DPPH radical-scavenging activity is defined as an activity unit and the DPPH radical-scavenging activity was expressed as U·g^−1^ FW.

Superoxide anion scavenging activity was measured by Nitrotetrazolium Blue chloride (NBT) reduction method [19]. 50% of the superoxide anion scavenging activity is defined as an activity unit. Superoxide anion scavenging activity was expressed as U·g^−1^ FW.

Hydroxyl radical-scavenging activity is determined by the method of Shon et al. [18]. 50% of the hydroxyl radical-scavenging activity is defined as an activity unit. Hydroxyl radical-scavenging activity was expressed as U·g^−1^ FW.

The total phenolic concentration of flesh extracts was measured using a modified Folin–Ciocalteu colorimetric method [20]. Absorbance was measured at 760 nm after 60 min at room temperature. The results were expressed as micrograms of gallic acid equivalents per gram of fresh weight.

The total flavonoid concentration of flesh extracts was determined using a colorimetric assay [20]. The absorbance of the solution versus a blank at 510 nm was measured after 60 min. The results were expressed as micrograms of catechin equivalents per gram of fresh weight.

### 2.7. Statistical Analysis

All data were analyzed by one-way analysis of variance (ANOVA) with SPSS 11.0 statistical software. Significant differences were performed by a least significant difference method (LSD test, *P* ≤ 0.05) for all treatments at different sampling times.

For multivariate analysis, data were centered and weighted by the inverse of the standard deviation of each variable in order to avoid dependence on measured units. Principal component analysis (PCA) and partial least squares (PLS) in this study were performed using Unscrambler 9.7 statistical software. Path analysis model was developed by DPS (v.8.01) software.

## 3. Results and Discussion

### 3.1. One-Way Analysis of Variance

1-Methylcyclopropene (1-MCP) has been proved to slow down the ripening of some fruits [21, 22]. In this work, the decay rate of blackberry fruit was reduced significantly (*P* < 0.05) by 1-MCP treatment (Table 1). The antioxidant enzymes, polyphenol oxidase (PPO), in 1-MCP treated fruit were also higher than control. On the other hand, a higher firmness was observed with vacuum precooling compared to control after 38 days of storage. The PPO activities were also enhanced by vacuum precooling after storage at 0°C for 38 days. Our finding suggested that both 1-MCP and vacuum precooling treatment had a potential value in delaying the senescence of blackberries.

The antioxidant activities can be characterized by TEAC, DPPH, FARP, and NBT radical-scavenging activities. Compared with vacuum precooling, at the end of storage, 1-MCP treated fruit had higher TEAC and DPPH radical-scavenging activities but lower FARP and NBT radical-scavenging activities. So, it was difficult to compare the effect between 1-MCP and vacuum precooling. For this reason, the PCA model was performed.

### 3.2. Principal Component Analysis

**Figure 1:**
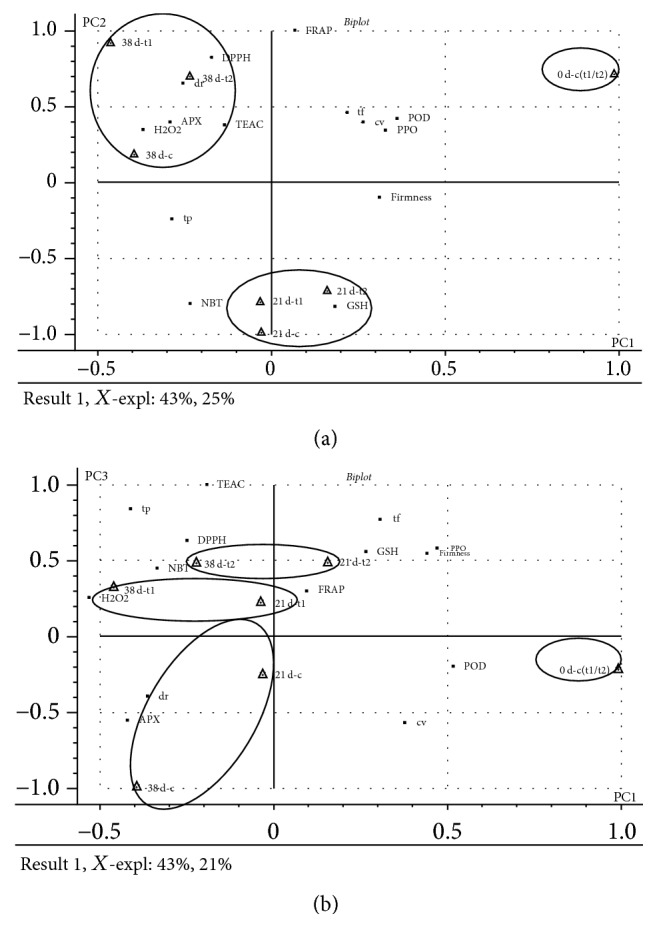
Loadings and scores from PCA of blackberries. “c”: the control group; “t1”: treatment of 1-MCP; “t2”: treatment of vacuum precooling; “dr”: decay rate; “cv”: hydroxyl radical-scavenging activity; “tp”: total phenol; “tf”: total flavonoids.

The parameters in Table 1 were used to develop the PCA model. The first three PCs explained 89% of the variance in the data, which was high enough to represent all the variables. The score plot for PC1 versus PC2 (Figure 1(a)) clearly distinguished three groups defined by length of storage, indicating that storage time had a major influence on the quality and reactive oxygen metabolism parameters of blackberry fruit.

Following PC3, the 1-MCP treated fruit and the vacuum precooled fruit were discriminated (Figure 1(b)). The loading plot of the variables showed that the TEAC and DPPH radical-scavenging activities had a heavy load on the positive coordinate of PC3. So, PC3 could be defined by antioxidant activities. The vacuum precooled fruit had higher positive scores for PC3 than 1-MCP treated fruit. These results suggested that precooled fruit might have values larger than the mean of the antioxidant activities, while 1-MCP treated fruit had relatively lower values.

### 3.3. Partial Least Squares

**Figure 2:**
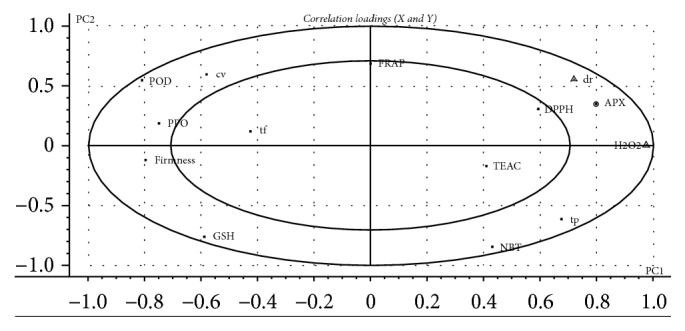
Correlation loading plot from a PLSR model. The inner and outer ellipses represent 50% and 100% of explained variance, respectively. “dr”: decay rate; “cv”: hydroxyl radical-scavenging activity; “tp”: total phenol; “tf”: total flavonoids.

H_2_O_2_ as signal molecule plays an important role inside plant bodies [23]. From the PCA study (Figure 1(a)), H_2_O_2_ content had a heavy load on the negative coordinate of PC1, suggesting that H_2_O_2_ content was the most important variable of blackberry fruit. We chose H_2_O_2_ content as *X* variable and the other parameters as *Y* variable to develop the PLS model to obtain a closer understanding of the relation between them. 64% of *X* variables explained 88% of the variability of *Y* variables. Many studies have showed that the production of H_2_O_2_ increased when plants were exposed to various biotic and abiotic stresses [23]. Peroxidase (POD) can decompose H_2_O_2_ by oxidation of cosubstrates, such as phenolic compounds and antioxidants [24], which could explain why a strongly negative correlation between POD activity and H_2_O_2_ content was found in the present study (Figure 2). It also showed that TEAC, FRAP, and NBT radical-scavenging activities slightly correlated with H_2_O_2_ (Figure 2), which indicated that they had little influence on H_2_O_2_ content.

### 3.4. Path Analysis

The direct effect of physiological parameters on H_2_O_2_ content was analyzed by PLS model. To find the indirect factors, the path analysis model was developed. As shown in Table 2, the indirect path coefficient of GSH based on DPPH was −0.3397, which suggested that GSH was also a factor affecting H_2_O_2_ content.

## 4. Conclusion

Based on the results of PCA, vacuum precooling treatment could play a stronger role in keeping the antioxidant activities of blackberry fruit than did the 1-MCP treatment. The score of PCA also revealed that H_2_O_2_ was the most important variable of blackberry fruit. Results from partial least squares regression and path analysis showed that POD activity had a direct effect and GSH content had an indirect effect on H_2_O_2_ content, while TEAC, FRAP, and NBT radical-scavenging activities had little effect on H_2_O_2_ content.

## Figures and Tables

**Table 1:** Changes of quality and reactive oxygen metabolism indexes in blackberries during postharvest storage with different treatments.

	Days of storage						
	Control			1-MCP		Vacuum precooling	
	Harvest	21 d	38 d	21 d	38 d	21 d	38 d
Firmness	21.38^d^	12.33^b^	6.09^a^	15.00^bc^	5.84^a^	17.76^bcd^	18.31^cd^
Decay rate (%)	0.00^a^	0.00^a^	21.67^c^	0.00^a^	11.67^b^	0.00^a^	18.34^c^
H_2_O_2_ (mg/100 g fw)	6.28^a^	16.76^b^	23.52^d^	18.92^c^	31.76^f^	16.99^b^	26.22^e^
GSH (mg/100 g fw)	61.55^d^	65.89^e^	26.62^a^	80.80^f^	39.43^c^	97.02^g^	35.22^b^
APX (U/g fw)	0.02^a^	0.03^a^	0.09^c^	0.05^ab^	0.08^bc^	0.02^a^	0.04^a^
POD (U/g fw)	105.71^b^	5.58^a^	1.32^a^	5.41^a^	4.17^a^	5.09^a^	4.96^a^
PPO (U/g fw)	1.54^f^	0.75^b^	0.59^a^	0.92^c^	0.91^c^	1.2^e^	1.1^d^
FRAP (U/g fw)	45.43^e^	30.04^b^	36.30^c^	28.03^a^	42.50^d^	36.86^c^	48.96^f^
TEAC (U/g fw)	0.2232^b^	0.1980^a^	0.2172^b^	0.2609^c^	0.3192^f^	0.2937^e^	0.2824^d^
DPPH (U/g fw)	31.74^c^	30.51^a^	31.24^b^	31.73^c^	38.30^e^	31.22^b^	36.61^d^
NBT (U/g fw)	7.80^a^	33.18^e^	26.18^c^	35.69^f^	24.25^b^	36.96^g^	30.77^d^
Hydroxyl radical- scavenging activity (U/g fw)	42.51^f^	38.17^a^	40.75^e^	38.90^b^	39.33^c^	39.97^d^	38.05^a^
Total phenol (*μ*g/g)	6.15^a^	8.43^c^	7.68^b^	9.02^d^	9.43^f^	9.18^e^	9.37^f^
Total flavonoid (*μ*g/g)	9.93^f^	8.15^b^	7.28^a^	9.18^d^	9.37^e^	8.57^c^	9.01^d^

The different superscript letters in the same row indicated significant difference (*P* < 0.05).

**Table 2:** The result of path analysis taking H_2_O_2_ as dependent variable.

	GSH	APX	POD	DPPH	Total flavonoid
Direct	0.0441	0.2559	−0.4243	0.6499	−0.1618
→GSH		−0.0307	0.0037	−0.0231	0.0101
→APX	−0.178		−0.116	0.0959	−0.1044
→POD	−0.0359	0.1923		0.0792	−0.2541
→DPPH	−0.3397	0.2436	−0.1213		0.2654
→Total flavonoid	−0.0371	0.066	−0.0969	−0.0661	
